# SOC stocks prediction on the basis of spatial and temporal variation in soil properties by using partial least square regression

**DOI:** 10.1038/s41598-023-34607-9

**Published:** 2023-05-16

**Authors:** Jawaria Usman, Shaheen Begum

**Affiliations:** Department of Environmental Sciences, Fatima Jinnah Women University, Rawalpindi, 46000 Pakistan

**Keywords:** Biogeochemistry, Climate sciences, Environmental sciences

## Abstract

Global warming is a wide-scale problem and soil carbon sequestration is its local scale, natural solution. Role of soil as carbon sink has been researched extensively but the knowledge regarding the role of soil variables in predicting soil carbon uptake and its retention is scarce. The current study predicts SOC stocks in the topsoil of Islamabad-Rawalpindi region keeping the soil properties as explanatory variables and applying the partial least square regression model on two different seasons’ datasets. Samples collected from the twin cities of Islamabad and Rawalpindi were tested for soil color, texture, moisture-content, SOM, bulk density, soil pH, EC, SOC, sulphates, nitrates, phosphates, fluorides, calcium, magnesium, sodium, potassium, and heavy metals (nickel, chromium, cadmium, copper and manganese) by applying standard protocols. Afterwards, PLSR was applied to predict the SOC-stocks. Although, current SOC stocks, ranged from 2.4 to 42.5 Mg/hectare, but the outcomes of PLSR projected that if soil variables remain unaltered, the SOC stocks would be likely to get concentrated around 10 Mg/hectare in the region. The study also identified variable importance for both seasons’ datasets so that noisy variables in the datasets could be ruled out in future researches and precise and accurate estimations could be made.

## Introduction

At global scale, there is a noticeable level of interest growing towards better management of soils’ organic carbon not only for dealing with the food security problems but also for tackling the changing climate. The major initiatives addressing this problem include 4p1000 initiative, REDD + and Global assessment of SOC sequestration potential program (GSOCseq)^1–3^.

Soil is considered to be the greatest of all sinks for fixing the atmospheric carbon. They hold double the amount of carbon as compared to the terrestrial vegetation^4,5^. Soil holds carbon content in the form of soil organic carbon (apart from the calcareous soils)^6,7^. The uptake of carbon in soil, commonly referred to as carbon sequestration occurs either directly when CO_2_ is transformed into inorganic compounds like calcium and magnesium carbonates by means of particular inorganic chemical reactions^8^; or it occurs indirectly when the biomass gets degraded and becomes part of the soil system in the form of organic compounds, broadly referred to as soil organic matter consisting of soil organic carbon along with other organic substances such as humus^9^. Most of the structural and functional aspects of soil system such as moisture retention, complex formation with the metal ions and the cation exchange capacity of the soils are dependent on the soil organic carbon^10,11^. But the impact of SOC on soil is not unidirectional. Soil properties also influence the capture, quality, distribution and retention time of SOC depending on various external and internal factors such as land use category and seasonal fluctuations in temperature and moisture^12,13^.

Uptake and retention of atmospheric carbon in soil is a complex phenomenon. This intricate process involves multiple variables belonging to all the spheres (atmosphere, biosphere, hydrosphere and lithosphere) of the ecosystem^14,15^.

Within the soil regime, the spatial and temporal changes in soil organic carbon stocks is very much dependent on the innate properties of soils. However, the statistics on the distribution of soil organic carbon (SOC) stocks in the soil profiles in relation to the soil variables are barely adequate.

Following research provides a holistic picture of different variables shaping the SOC stocks in the top soils of Islamabad-Rawalpindi region.

## Methodology

For present research, 204 samples (including field replicates) were collected from the topsoil (0–30 cm) of Islamabad and Rawalpindi region. Atmospheric temperature and elevation were noted at the field along with the GPS readings and soil properties were assessed at Fatima Jinnah Women university laboratory. With the intent of studying the spatial variation, the samples were collected from three different land use types i.e., agricultural fields, urban lands (public parks) and forest area of Islamabad and Rawalpindi region. In order to assess the temporal change, the samples were collected in two seasons i.e., summer and winter. The samples were analyzed for physical soil variables such as soil color which was assessed by using Munsell soil color chart^16,17^; soil texture that was assessed by hydrometer method^17^; soil moisture content which was calculated by using loss on drying method^17^; organic matter that was assessed by loss on ignition technique^18,19^ and soil bulk density which was assessed by means of compliant cavity method^20^. Chemical parameters were also analyzed by using standard protocols. pH and EC were measured by using Crison MM40 portable multimeter kit^17^. Soil organic carbon was assessed by modified Walky and Black method^21^; soil sulphates, nitrates, phosphates were analyzed by using UV/VIS spectrophotometry^17^; fluorides were assessed by Ion selective electrode^22^. Sodium and potassium ions were analyzed by flame photometry and calcium content, magnesium content and heavy metals like nickel, chromium, cadmium, copper and manganese were assessed by means of atomic absorption spectrophotometry^17^.

Afterwards, samples were subjected to statistical analysis. Partial Least square regression statistical model was applied on both seasons’ (summer and winter) datasets. These predictive statistics were performed through XLStat. Precisely, in the two datasets, partial least square regression model was applied in order to predict the SOC stocks of the soils of the region based on the aforementioned independent variables as well as the current SOC stocks in the top layer soil. PLSR was applied on the two seasons’ datasets separately in order to identify the unique variables impacting the respective seasons data.

## Results and discussion

Multiple researches have been conducted on estimation of level, quality and distribution pattern of the SOC stocks all around the globe^23–25^. But the data on the factors reshaping these stocks is scarce. In recent times, management of soil organic carbon has been considered as one of the major and efficiently exploitable tools combating the rising greenhouse gas emissions (particularly carbon emissions). There are a number of factors governing the uptake, retention and variability in the behavior of soil organic carbon^14^. Amongst the other controlling factors such as climatic conditions, hydrological regime, biomass input and land use variation, the one major entity/system that consistently affects the SOC uptake as well as its storage is the soil regime. Soil variables, as a unit, can be regarded as one of the most significant and decisive factors in either enhancing or regressing the entire process of carbon uptake and its preservation.

Availability of comprehensive knowledge regarding the impact of soil variables on SOC stocks is undoubtedly a vital prerequisite for increasing the rate of carbon sequestration in a particular area.

Partial least square regression is a predictive statistical model which projects the dependent variable on the basis of single or multiple independent variables. In present research, the current soil properties were addressed as the independent variables and the SOC stocks were addressed as dependent variable. On the basis of the whole summer data distribution, the one datum i.e., S23 was selected as the validation dataset. The summary statistics of the summer and winter dataset along with their respective validation datasets are provided in Tables 1 and 2 respectively. During summer season, SOC Stock in topsoil of study area ranged from 2.352 Mg/hectare to 42.453 Mg/hectare with a mean value of 11.131 and standard deviation of 7.391.

**Table 1:** Summary statistics summer dataset.

Variable	Minimum	Maximum	Mean	Std. deviation	Summary statistics of validation set
SOC Stock	2.352	42.453	11.131	7.391	8.668
Temperature	23.000	34.000	27.720	2.540	27.000
Elevation	436.000	1096.000	577.780	149.556	536.000
Clay	0.000	23.250	5.793	5.575	6.250
Silt	5.000	50.000	20.546	12.443	13.750
Sand	31.600	89.525	70.612	13.468	73.575
MC	1.652	27.150	11.366	7.513	9.981
OM	0.160	2.650	0.705	0.426	0.510
BD	0.769	4.660	1.924	0.878	1.833
pH	6.650	8.950	7.697	0.640	7.920
EC	54.000	516.500	225.18	114.975	245.800
SOC	0.059	0.414	0.189	0.076	0.158
Sulphates	0.070	4.662	1.074	1.209	0.153
Nitrates	0.055	9.467	2.429	2.960	4.980
Phosphates	3.596	16.747	8.670	3.116	9.094
Fluoride*	0.187	3.885	0.957	0.642	0.578
Ca	703.539	2462.429	1247.5	422.086	1981.907
Mg	6.500	15.327	13.683	2.694	14.569
Na	20.000	54.500	31.200	8.327	26.500
K	21.500	204.500	61.520	46.626	34.500
Ni	2.288	8.157	4.564	1.206	4.018
Cr	0.060	1.387	0.394	0.251	0.264
Cd	0.168	0.925	0.530	0.205	0.750
Cu	0.171	0.740	0.435	0.121	0.323
Mn	2.911	7.853	4.503	0.839	4.343

*^26^.

**Table 2:** Summary statistics winter dataset.

Variable	Minimum	Maximum	Mean	Std. deviation	Summary statistics of validation set
SOC Stock	2.496	20.751	8.507	3.650	3.789
Temperature	7.000	13.000	9.450	1.648	9.000
Elevation	436.000	1096.000	579.660	148.400	442.000
Clay	0.000	21.250	7.100	5.141	6.250
Silt	2.500	60.000	23.750	11.998	28.750
Sand	33.900	91.625	68.317	14.031	64.663
MC	0.367	29.631	13.933	6.978	9.302
OM	0.084	1.161	0.405	0.197	0.270
BD	0.795	2.796	1.559	0.434	1.561
pH	7.730	10.275	9.063	0.655	8.765
EC	75.550	455.500	153.970	80.122	77.050
SOC	0.079	0.387	0.183	0.068	0.115
Sulphates	0.019	7.977	1.769	1.768	0.575
Nitrates	0.019	2.835	0.551	0.582	0.229
Phosphates	5.298	63.741	21.454	19.889	41.890
Fluoride*	0.476	5.586	2.093	1.298	0.822
Ca	174.960	1767.878	908.748	440.377	806.663
Mg	2.656	10.286	6.121	1.980	5.016
Na	27.000	90.000	58.390	12.945	65.500
K	15.500	111.000	38.470	19.641	23.500
Ni	2.460	6.752	4.641	1.042	3.407
Cr	0.009	1.975	0.613	0.519	0.137
Cd	0.115	0.930	0.406	0.196	0.105
Cu	0.031	1.046	0.445	0.167	0.263
Mn	2.102	5.964	4.352	0.754	4.015

*^26^.

### Predictions and residuals

On the basis of the explanatory datasets of each season, the SOC stock predictions were made. The detailed datasets along with the standardized residuals for each season i.e., summer and winter are given in Tables 3 and 4 respectively. For majority of the observations, the predicted SOC stock values were found to be lower than the current SOC stock values.

**Table 3:** Predictions and residuals (Variable SOC Stock) summer season.

Observation	Weight	SOC Stock	Pred (SOC Stock)	Residual	Std. residual	Std. dev. on pred. (Mean)	Lower bound 95%(Mean)	Upper bound 95%(Mean)	Std. dev. on pred. (Observation)	Lower bound 95% (Observation)	Upper bound 95% (Observation)
S1	1	12.853	10.906	1.947	0.269	0.546	9.809	12.003	3.858	3.154	18.658
S2	1	12.257	10.637	1.620	0.224	0.547	9.537	11.737	3.858	2.884	18.390
S3	1	9.014	9.631	− 0.617	− 0.085	0.560	8.505	10.758	3.860	1.875	17.388
S4	1	8.321	8.788	− 0.467	− 0.065	0.581	7.620	9.956	3.863	1.026	16.551
S5	1	9.589	9.396	0.193	0.027	0.565	8.260	10.532	3.860	1.638	17.154
S6	1	21.640	16.051	5.589	0.772	0.689	14.666	17.436	3.881	8.253	23.849
S7	1	5.529	3.991	1.538	0.213	0.819	2.345	5.637	3.906	− 3.858	11.840
S8	1	6.337	4.847	1.490	0.206	0.766	3.308	6.386	3.895	− 2.980	12.674
S9	1	7.013	5.447	1.566	0.216	0.731	3.978	6.916	3.888	− 2.366	13.261
S10	1	42.453	32.745	9.707	1.341	1.928	28.870	36.621	4.278	24.148	41.342
S11	1	21.308	19.771	1.538	0.212	0.919	17.924	21.617	3.928	11.877	27.664
S12	1	7.244	11.471	− 4.227	− 0.584	0.546	10.373	12.569	3.858	3.719	19.224
S13	1	4.690	6.870	− 2.180	− 0.301	0.656	5.551	8.188	3.875	− 0.917	14.656
S14	1	13.768	20.134	− 6.366	− 0.879	0.944	18.237	22.031	3.934	12.229	28.039
S15	1	7.027	7.665	− 0.638	− 0.088	0.621	6.417	8.913	3.869	− 0.110	15.440
S16	1	15.590	14.179	1.411	0.195	0.605	12.964	15.394	3.866	6.409	21.949
S17	1	6.691	4.551	2.140	0.296	0.784	2.975	6.126	3.898	− 3.283	12.385
S18	1	14.499	16.572	− 2.072	− 0.286	0.717	15.131	18.013	3.886	8.763	24.380
S19	1	6.936	9.098	− 2.162	− 0.299	0.573	7.947	10.248	3.862	1.338	16.858
S20	1	8.156	9.486	− 1.330	− 0.184	0.563	8.354	10.618	3.860	1.729	17.243
S21	1	8.435	9.058	− 0.623	− 0.086	0.574	7.905	10.211	3.862	1.298	16.819
S22	1	10.353	14.891	− 4.538	− 0.627	0.633	13.618	16.164	3.871	7.112	22.670
S24	1	17.865	17.583	0.282	0.039	0.776	16.023	19.143	3.897	9.752	25.414
S25	1	8.627	11.599	− 2.972	− 0.411	0.547	10.500	12.699	3.858	3.847	19.352
S26	1	6.045	6.720	− 0.674	− 0.093	0.663	5.387	8.053	3.876	− 1.069	14.509
S27	1	7.209	7.857	− 0.649	− 0.090	0.613	6.625	9.090	3.868	0.085	15.630
S28	1	7.301	7.230	0.072	0.010	0.640	5.944	8.515	3.872	− 0.552	15.011
S29	1	4.102	9.874	− 5.772	− 0.797	0.556	8.756	10.991	3.859	2.119	17.629
S30	1	7.326	7.973	− 0.647	− 0.089	0.609	6.749	9.196	3.867	0.201	15.744
S31	1	6.186	8.317	− 2.131	− 0.294	0.596	7.119	9.515	3.865	0.550	16.084
S32	1	10.144	10.831	− 0.688	− 0.095	0.546	9.734	11.929	3.858	3.079	18.584
S33	1	11.464	18.724	− 7.259	− 1.003	0.848	17.019	20.429	3.912	10.862	26.585
S34	1	9.053	15.127	− 6.074	− 0.839	0.644	13.833	16.421	3.873	7.344	22.910
S35	1	22.678	18.935	3.742	0.517	0.862	17.202	20.668	3.915	11.068	26.803
S36	1	10.126	13.289	− 3.162	− 0.437	0.576	12.131	14.446	3.862	5.528	21.050
S37	1	15.740	15.797	− 0.057	− 0.008	0.676	14.439	17.156	3.878	8.004	23.591
S38	1	9.156	11.298	− 2.142	− 0.296	0.546	10.201	12.394	3.858	3.545	19.050
S39	1	8.002	6.552	1.450	0.200	0.672	5.202	7.901	3.877	− 1.240	14.344
S40	1	22.711	13.136	9.575	1.323	0.572	11.986	14.285	3.861	5.376	20.895
S41	1	20.323	17.863	2.460	0.340	0.793	16.268	19.457	3.900	10.025	25.701
S42	1	28.049	22.796	5.253	0.726	1.138	20.510	25.082	3.985	14.789	30.804
S43	1	18.935	21.273	− 2.338	− 0.323	1.025	19.213	23.333	3.954	13.327	29.219
S44	1	5.749	10.824	− 5.075	− 0.701	0.546	9.727	11.922	3.858	3.072	18.577
S45	1	4.240	2.733	1.507	0.208	0.902	0.920	4.546	3.924	− 5.153	10.619
S46	1	9.546	8.861	0.685	0.095	0.579	7.698	10.025	3.862	1.099	16.623
S47	1	7.708	10.867	− 3.160	− 0.437	0.546	9.770	11.965	3.858	3.115	18.620
S48	1	2.352	4.223	− 1.871	− 0.258	0.804	2.607	5.840	3.903	− 3.620	12.066
S49	1	4.465	0.368	4.097	0.566	1.070	− 1.783	2.519	3.966	− 7.602	8.338
S50	1	9.181	0.834	8.347	1.153	1.036	− 1.249	2.917	3.957	− 7.118	8.786
S51	1	2.556	− 1.127	3.683	0.509	1.182	− 3.503	1.249	3.998	− 9.161	6.907
Predictions and residuals (Variable SOC Stock):											
S23	1	8.668	14.750	− 6.082	− 0.840	0.546	13.653	15.847	3.858	6.998	22.502

The predictions and residuals corresponding to the observations of the validation set are displayed in the second part of the table.

**Table 4:** Predictions and residuals (Variable SOC Stock) winter season.

Observation	Weight	SOC Stock	Pred(SOC Stock)	Residual	Std. residual	Std. dev. on pred. (Mean)	Lower bound 95%(Mean)	Upper bound 95%(Mean)	Std. dev. on pred. (Observation)	Lower bound 95% (Observation)	Upper bound 95% (Observation)
W1	1	2.990	2.991	− 0.001	0.000	0.704	1.577	4.405	2.389	− 1.810	7.792
W2	1	6.727	3.536	3.191	0.893	0.650	2.231	4.842	2.374	− 1.234	8.307
W3	1	5.649	4.080	1.570	0.439	0.597	2.879	5.280	2.360	− 0.663	8.822
W4	1	2.496	3.305	− 0.809	− 0.226	0.672	1.954	4.657	2.380	− 1.478	8.088
W5	1	6.110	5.298	0.812	0.227	0.488	4.318	6.279	2.335	0.607	9.990
W6	1	6.525	4.981	1.544	0.432	0.515	3.946	6.016	2.341	0.278	9.685
W8	1	6.910	5.220	1.690	0.473	0.494	4.227	6.214	2.336	0.526	9.915
W9	1	4.657	4.779	− 0.121	− 0.034	0.533	3.708	5.850	2.345	0.067	9.490
W10	1	7.819	6.459	1.360	0.381	0.400	5.655	7.263	2.318	1.801	11.117
W11	1	8.469	8.214	0.255	0.071	0.328	7.555	8.873	2.307	3.579	12.849
W12	1	7.606	7.688	− 0.082	− 0.023	0.339	7.007	8.370	2.308	3.050	12.327
W13	1	9.432	8.210	1.222	0.342	0.328	7.551	8.869	2.307	3.575	12.846
W14	1	5.725	7.351	− 1.627	− 0.455	0.351	6.645	8.058	2.310	2.709	11.994
W15	1	3.106	5.902	− 2.796	− 0.782	0.439	5.019	6.785	2.325	1.230	10.575
W16	1	11.685	9.877	1.808	0.506	0.361	9.152	10.603	2.312	5.232	14.522
W17	1	7.697	8.978	− 1.282	− 0.358	0.330	8.314	9.642	2.307	4.342	13.614
W18	1	8.999	9.971	− 0.972	− 0.272	0.366	9.236	10.706	2.312	5.324	14.618
W19	1	5.568	7.558	− 1.990	− 0.557	0.343	6.868	8.248	2.309	2.918	12.198
W20	1	7.292	8.958	− 1.666	− 0.466	0.330	8.295	9.622	2.307	4.322	13.594
W21	1	13.04	10.189	2.855	0.799	0.377	9.430	10.947	2.314	5.538	14.839
W22	1	7.066	9.957	− 2.892	− 0.809	0.365	9.224	10.691	2.312	5.311	14.604
W23	1	5.395	6.927	− 1.532	− 0.428	0.372	6.179	7.674	2.313	2.278	11.575
W24	1	8.348	7.026	1.322	0.370	0.367	6.289	7.763	2.312	2.379	11.673
W25	1	5.874	5.794	0.081	0.023	0.448	4.894	6.694	2.327	1.118	10.469
W26	1	12.18	8.600	3.577	1.001	0.326	7.944	9.256	2.306	3.965	13.235
W27	1	6.895	5.807	1.088	0.304	0.447	4.909	6.705	2.326	1.132	10.482
W28	1	8.030	7.362	0.667	0.187	0.351	6.657	8.067	2.310	2.720	12.004
W29	1	8.161	7.472	0.689	0.193	0.347	6.775	8.168	2.309	2.831	12.113
W30	1	8.668	8.533	0.135	0.038	0.326	7.877	9.188	2.306	3.898	13.168
W31	1	9.072	8.666	0.407	0.114	0.327	8.009	9.322	2.306	4.031	13.301
W32	1	6.652	7.147	− 0.496	− 0.139	0.361	6.423	7.872	2.311	2.502	11.792
W33	1	9.657	9.053	0.604	0.169	0.332	8.386	9.720	2.307	4.417	13.690
W34	1	4.970	8.407	− 3.438	− 0.962	0.326	7.752	9.063	2.306	3.773	13.042
W35	1	13.23	12.699	0.530	0.148	0.575	11.543	13.855	2.355	7.967	17.431
W36	1	4.966	10.393	− 5.427	− 1.518	0.390	9.610	11.176	2.316	5.739	15.048
W37	1	8.165	11.105	− 2.940	− 0.822	0.439	10.223	11.987	2.325	6.433	15.777
W38	1	20.33	15.632	4.694	1.313	0.869	13.886	17.379	2.443	10.723	20.542
W39	1	20.75	14.266	6.485	1.814	0.728	12.803	15.729	2.396	9.450	19.082
W40	1	13.96	12.271	1.692	0.473	0.536	11.194	13.349	2.345	7.558	16.984
W41	1	8.942	12.186	− 3.244	− 0.908	0.529	11.124	13.248	2.344	7.477	16.896
W42	1	11.71	13.247	− 1.538	− 0.430	0.627	11.986	14.507	2.368	8.489	18.005
W43	1	8.606	9.201	− 0.596	− 0.167	0.335	8.527	9.875	2.308	4.564	13.839
W44	1	7.965	6.684	1.281	0.358	0.386	5.909	7.460	2.316	2.031	11.338
W45	1	5.039	8.980	− 3.941	− 1.102	0.331	8.316	9.644	2.307	4.344	13.616
W46	1	12.75	10.770	1.983	0.555	0.415	9.937	11.603	2.321	6.107	15.434
W47	1	8.812	8.353	0.459	0.128	0.327	7.697	9.010	2.306	3.718	12.988
W48	1	8.366	8.846	− 0.480	− 0.134	0.328	8.186	9.505	2.307	4.210	13.481
W49	1	9.170	12.423	− 3.252	− 0.910	0.550	11.318	13.527	2.348	7.703	17.142
W50	1	11.48	11.691	− 0.215	− 0.060	0.486	10.715	12.667	2.334	7.000	16.382
W51	1	11.66	12.327	− 0.665	− 0.186	0.541	11.240	13.415	2.346	7.612	17.043
Predictions and residuals (Variable SOC Stock):											
W7	1	3.789	5.343	− 1.554	− 0.435	0.704	3.929	6.757	2.389	0.542	10.144

The predictions and residuals corresponding to the observations of the validation set are displayed in the second part of the table.

### Factors of soil formation

The dynamics of SOM accumulation and stabilization differ in different types of soils^27^. Among other governing factors such as climate, microbiota and vegetation types; the pedogenic processes not only regulate the storage of SOM but also predict the behavior of SOM in long term. The innate structural properties of soil such as its texture and bulk density define how SOM will be retained within different soil horizons. Similarly, other soil formation processes i.e., deposition and removal of nutrients (illuviation and eluviation) define how SOM will get adsorbed onto mineral surfaces in both short as well as long run^28^. Furthermore, the physicochemical characteristics of soil also govern the permanence and stability of SOM.

The current research discusses these soil formation processes such as the innate physicochemical properties and the nutrient deficiencies (nitrates and sulphate deficiencies) within the alkaline soils of Islamabad and Rawalpindi in terms of variables of importance.

### Variable importance in the projection

**Figure 1:**
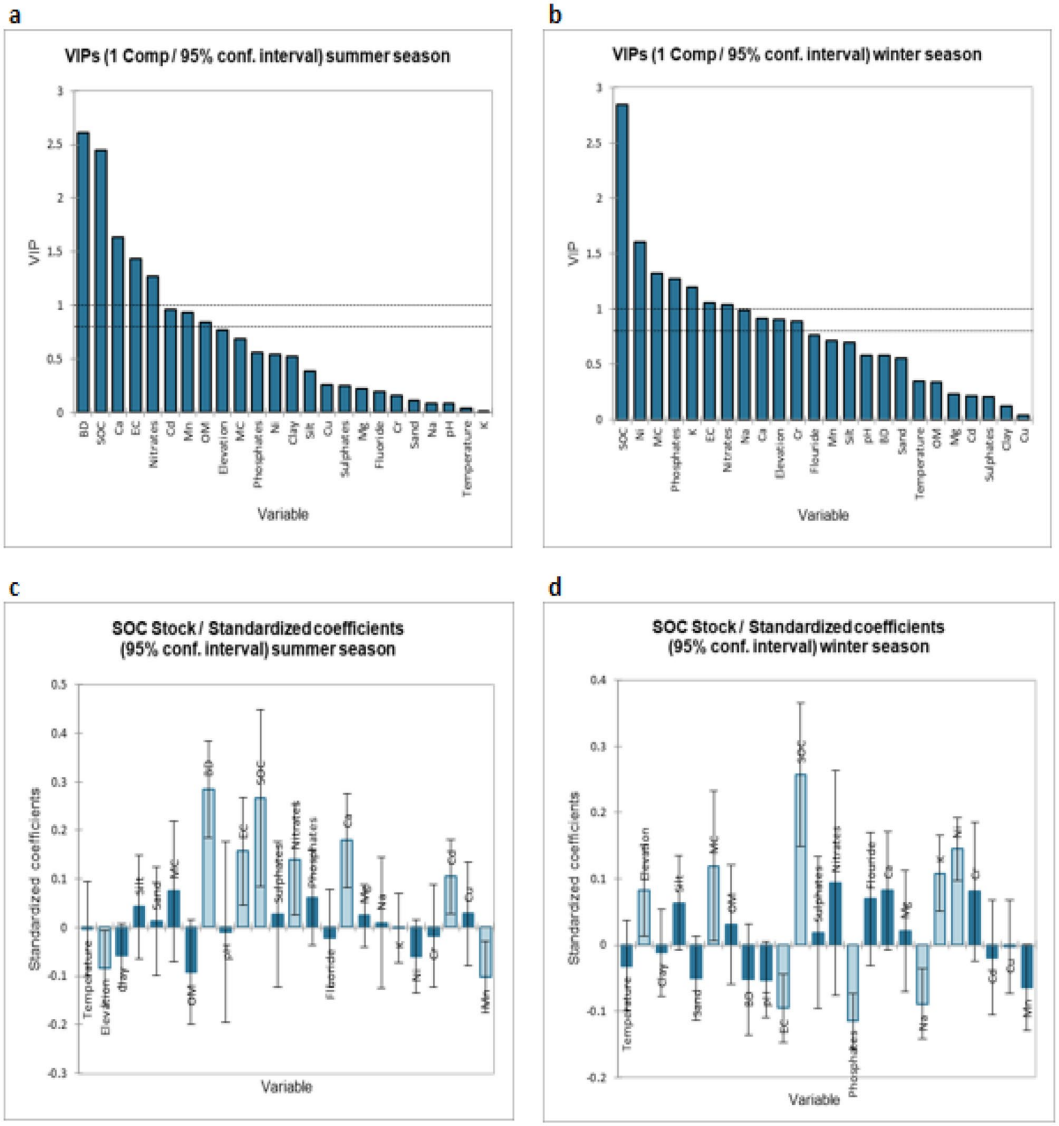
(**a**) Variable importance in the projection of summer dataset. (**b**) Variable importance in the projection of winter dataset. (**c**) SOC stock/Standardized coefficients for summer dataset. (**d**) SOC stock/Standardized coefficients for winter dataset.

As per the results of the summer dataset PLSR, the most important variables impacting the whole summer dataset were found to be bulk density, soil organic carbon, calcium ions, soil EC, and soil nitrates as shown in Fig. 1a. While the most important variables playing the key role in the winter season were soil organic carbon, nickel concentrations, moisture content of the soil, phosphate content, potassium ion, soil EC and soil nitrates as shown in Fig. 1b. The standardized coefficients of independent variables against the SOC stock as dependent variable for summer season and the winter season are shown in Fig. 1c,d.

Among the samples collected in the summer season, a huge number of samples i.e., 77% had bulk density value less than 2 g/cm^3^. And in winter dataset, a major percentage i.e., 82% of the whole data lied within the 1 g/cm^3^ to 2 g/cm^3^. These values are also in consensus with the available literature of the region i.e., Islamabad and Rawalpindi^29–31^. Thus, they can be considered as the predominantly prevailing bulk density values in the region.

The range of bulk density in the study area denotes a potential for increased SOC retention. Naturally, the bulk density consists of air spaces and the SOM. In the sandy-textured soils such as sandy loams of the study area, bulk density values greater that 1.7 g/cm^3^ hinders the natural root growth whereas in fine-textured soils this value further drops down to 1.5 g/cm^3^^32,33^. In terms of carbon storage, bulk density values less than 1.3 g/cm^3^ is considered to be as good, while greater than 1.8 g/cm^3^ is considered to be as very bad^32^. Besides soil texture, the nutrient concentration also leads to higher values of bulk density. Chaudhari et al. reported negative relationships of soil bulk density with soil silicates, soil calcium carbonate and total micro and macro nutrient contents^34^. Hence soils of Islamabad and Rawalpindi should be managed for lowering the bulk density.

Within the soils of Islamabad and Rawalpindi, soil organic carbon in both the seasons majorly lied from 0.16 to 0.25%. Majority of the soil samples had SOC percentage less than 0.25%. Around 47% of the samples in the summer dataset and 45% in the winter dataset had SOC ranging from 0.16% to 0.25%.

SOC values less than 2% indicate that the soil is of poor-quality in terms of its structural stability^35^. In agricultural criterion too, the soils having less than 2% SOC value are widely considered to be the ones that have more chances to get deteriorated in terms of productivity and yield^36^. In the dry land cropping system, even no-tillage doesn’t work alone to cause increase in the SOC percentage^37–39^. The dry-aggregates associated carbon is the prime SOC stock stored in the semi-arid soils of Pakistan^40^. So, in order to increase it, the focus should be shifted to broader soil management strategies such as integrated nutrient management and planned planation.

Majority of the samples (57% in the summer and 35% in the winter) lied in the range of 1000 ppm to 1500 ppm of calcium content in both the seasons. Available research also supports the data ranges^41^. In comparison to the calcium carbonate content, the exchangeable calcium plays an important role in the protection of SOC. However, in the presence of adequate nutrients especially organic amendments in the form of compost, the calcium carbonate is readily converted to the exchangeable calcium. This change within the soils of a region can further enhance the process of organo-calcium complex formation and thus can play a key role in long term preservation of soil organic carbon^42–44^.

For both the seasons, most of the samples had nitrate concentration less than 3 ppm. In summer season dataset, a major percentage of samples i.e., 71% lied from 0 to 3 ppm. While in winter season, 88% of the samples had nitrates level less than 1 ppm. Within the winter dataset, the number of samples continuously decreased with an increase in the nitrate content. Shaheen, 2016 also described the soils of Rawalpindi region to be nitrate deficient. The possible reasons for this deficiency might be leaching because of the coarse-textured soils^45^ of the region and volatilization^46^ due to alkaline pH of the soils as well as the increasing temperatures^47–49^.

The minimum required soil nitrate concentration available for the plants is 10 ppm (preplant season) to 30 ppm (growing season)^50^. Whereas soils of Pakistan are deficient in nitrates^51,52^.

Due to its high mobility, the nitrate ion does not get adsorbed at the cation exchange site within the soils. Hence, it is readily lost, especially from the calcareous soils such as those of Pakistan^53,54^. This loss not only affects the quality of the soil but it also translates into economic loss in terms of annual crop production^55–57^. Nitrogen use efficiency within the Pakistani agriculture system rarely go beyond 40%. Mostly (around 22–53%) of the nitrogen content added is lost in the form of ammonia, due to alkaline nature of soils rising temperature. The other factors that contribute to this volatilization are low moisture content and salinity. This volatilization can be controlled up to 80% by adopting good soil management practices^52^.

Phosphate content for both the seasons majorly lied from 5 to 100 ppm. The summer dataset majorly concentrated from 5.1 ppm to 10 ppm with having 61% of the samples from the whole data. And in winter season, major percentage of samples i.e., 61% lied from 0.1 ppm to 15 ppm. The phosphate concentration ranges also coincided with the available studies of the region^58,59^.

According to the Minimum Levels for Sustainable Nutrition (MSLN) guidelines, phosphate range in healthier soils is 7 ppm to 50 ppm. So, the levels of phosphates within the soils of Islamabad and Rawalpindi are satisfactory^60,61^.

Nickel in both seasons’ datasets lied under 6 ppm. In the summer dataset, about 80% samples lied in the range of 3.1 ppm to 6 ppm. In winter season, the highest percentage of samples i.e., 35% lied in the range of 5.1 ppm to 6 ppm. Results of the present study lied close to nickel concentrations reported by Ashraf et al. 2019 for the study region^62^.

Presence of nickel ions is crucial for the soil organic matter. In alkaline soils having pH values higher than 8, the formation of Ni-calcite complex leads to the protection of organic matter^63,64^. In a recent in-situ research, nickel nanoparticles were used to enhance the mineralization process of CO_2_ by using brines to permanently sequester the impure carbon dioxide into carbonates^65^. However, in ex-situ environment, role of Ni for the sequestration has not been studied in-depth lately. In 1979, research was conducted to assess the role of nickel as an oxide in the sandy soils of different pH ranges on mineralization of carbon. This research concluded that the mineralization diminished with increasing nickel concentration, however, at their highest soil pH i.e., 7.6, the extent of this mineralization was not the same as that of the lower pH soils^66^. So, there is potentially a research gap that needs to be addressed regarding the role of nickel in carbon sequestration.

Potassium content for majority of the samples (i.e., 67%) in summer season, lied in the range of 1 ppm to 55 ppm. Around 22% of the samples lied in the concentration bracket of 56 ppm to 110 ppm. In the winter dataset, 75% of the samples lied in the range of 1 ppm to 40 ppm. The number of samples decreased with an increase in the concentration of potassium during both seasons. The data range also coincides with the available literature^41,67–69^. Potassium content is predominantly high within the soils of most of Pakistani regions including the region of Pothwar which has mica in the parent rock material^70^. The alkaline soils having high sodium and potassium values should be irrigated with low-salt water^71^. The other soil factors associated with the regulation of potassium ion are the charge balance and the enzymatic activity regulation within the soils’ microbial population as well as the higher plants^72,73^. It also affects the moisture content of the soil by regulating the osmotic uptake in plants, thus indirectly, it plays an important part in the carbon retention within the soil^74,75^.

### SOC stock prediction

**Figure 2:**
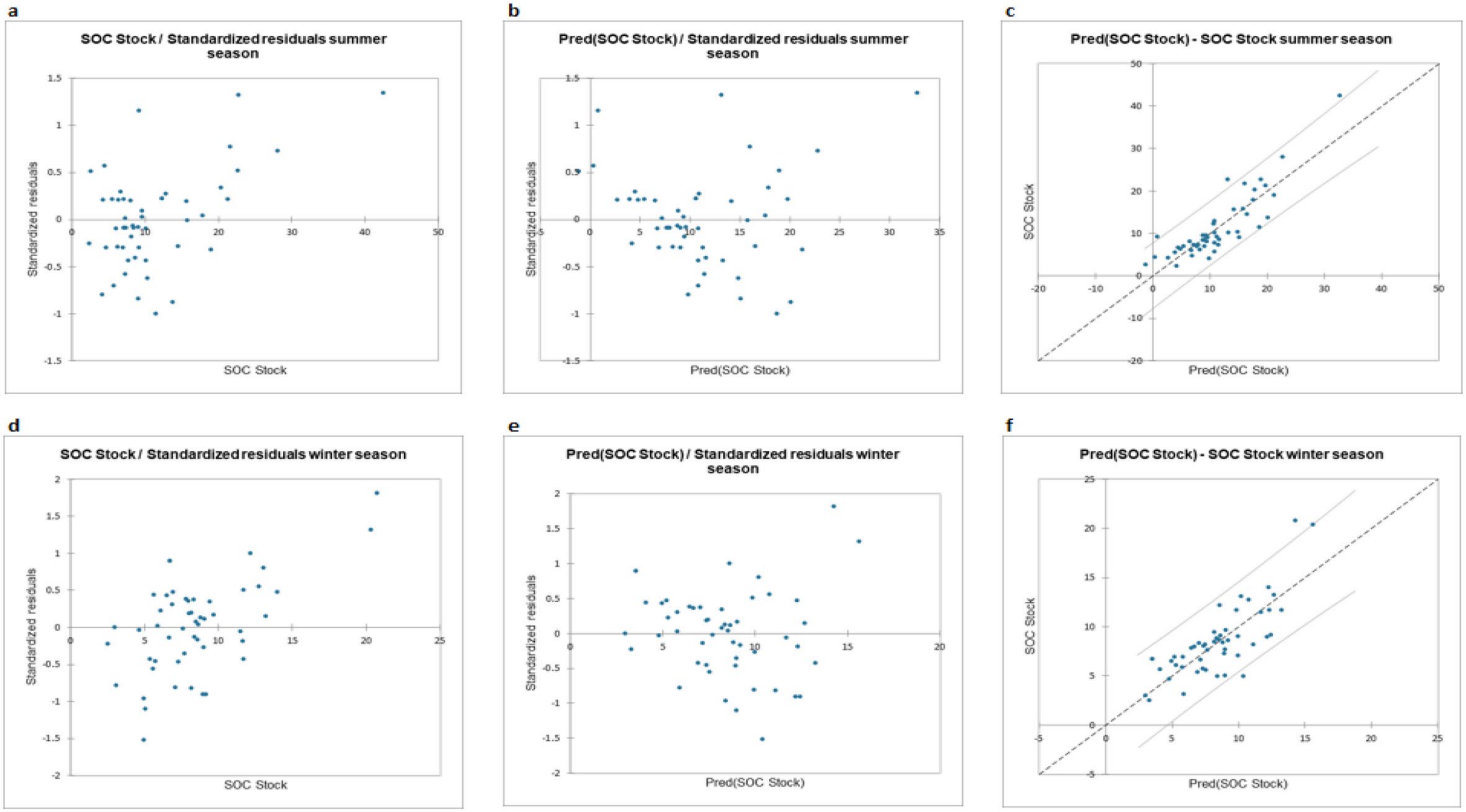
(**a**) SOC stock/standardized residuals summer season. (**b**) Predicted SOC stock/Standardized residuals summer season. (**c**) Predicted SOC stock—Current SOC stock summer season. (**d**) SOC stock/standardized residuals winter season. (**e**) Predicted SOC stock/Standardized residuals winter season. (**f**) Predicted SOC stock—Current SOC stock winter season.

The current SOC stock values and the predicted SOC stock values for both the seasons are provided in Fig. 2. At first step of PLSR, SOC stocks were plotted against the standardized residuals. Although the summer SOC stock upper range was identified around 40 Mg/hectare, which coincided with the available literature for the region^76^ still most of values of current SOC stock were concentrated below 20 Mg/hectare in summer dataset and 10 Mg/hectare in winter dataset as shown in Fig. 2a,d. Then, based on the standardized residual values the SOC predictions were made which are given in Fig. 2b,e for summer and winter season respectively. According to the PLSR outcome, majority of the predicted SOC stock values were found to be concentrated around 10 Mg/hectare as shown in Fig. 2c,f for summer and winter season datasets respectively.

The major probable reasons (in terms of soil properties) behind such a low level of SOC stock values might be the higher bulk density values, low nitrate concentrations as depicted in the variable importance (Fig. 1a,b).

### Outliers analysis

**Figure 3:**
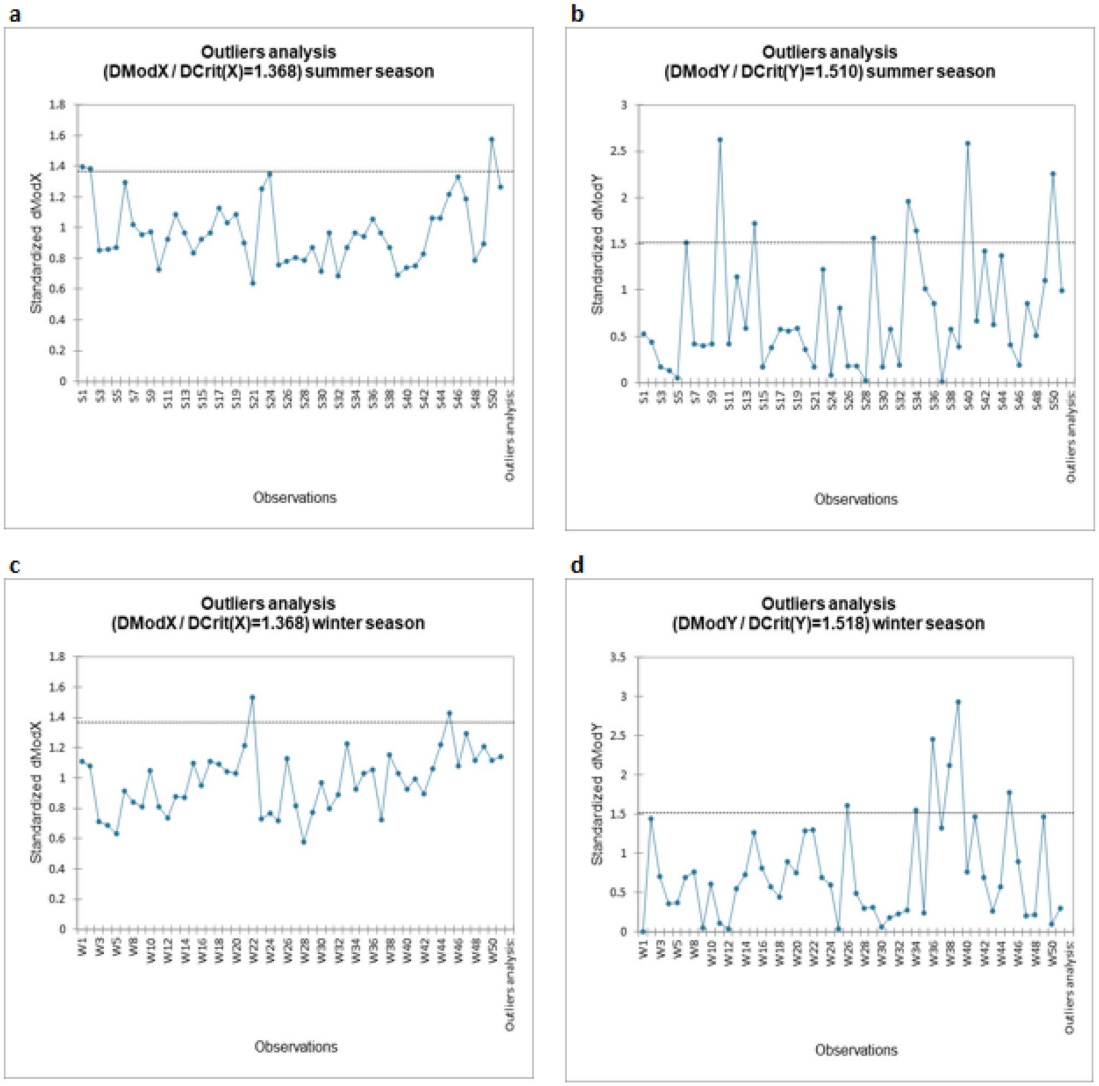
(**a**) Outliers analysis of observations on the basis of explanatory variable i.e., soil properties summer season. (**b**) Outliers analysis of the observations on the basis of dependent variables i.e., SOC stocks summer season. (**c**) Outliers analysis of observations on the basis of explanatory variable i.e., soil properties winter season. (**d**) Outliers analysis of the observations on the basis of dependent variables i.e., SOC stocks winter season.

Outliers’ analysis results are provided in Fig. 3. The distance to model (DMoD) values for both the summer and winter dataset was found to be 1.368 for the explanatory variable as shown in Fig. 3a,c. And the DMoD for the dependent variable i.e., SOC stock was found to be 1.510 for the summer dataset (Fig. 3b) and 1.518 for the winter dataset (Fig. 3d).

## Conclusion and recommendations

According to the outcomes of the partial least square regression, it can be concluded that if the current status of soil variables prolongs unaltered, the SOC stocks of the topsoil of Islamabad and Rawalpindi region will be likely to deteriorate further. However, if the variables identified as most important in both season’s datasets (such as bulk density, soil nitrates and moisture content) are managed at the regional level, the SOC stock would likely to improve and thus could play a major role in mitigating the impacts of climate change particularly the warming of this region.

Some of the recommendations for enhancing carbon stocks in alkaline soils of Islamabad and Rawalpindi are as follows.

- In order to increase soil carbon stocks, integrated nutrient management approaches can be applied.
- The alkaline soils of the region should be irrigated with low-salt water in order to naturally optimize their alkalinity.
- The coarse-textured, alkaline soils of Islamabad and Rawalpindi are deficient in the nitrate content. This deficiency can directly attribute to the decreasing soil carbon stocks. Hence, nitrate content of the soil must be managed in order to improve the current soil carbon stocks.

## Data Availability

The data that supports the findings of this study are available from the corresponding author upon reasonable request.
